# Mesenchymal Stem Cell and Hematopoietic Stem and Progenitor Cell Co-Culture in a Bone-Marrow-on-a-Chip Device toward the Generation and Maintenance of the Hematopoietic Niche

**DOI:** 10.3390/bioengineering11080748

**Published:** 2024-07-24

**Authors:** Dionysia Kefallinou, Maria Grigoriou, Dimitrios T. Boumpas, Angeliki Tserepi

**Affiliations:** 1 Institute of Nanoscience and Nanotechnology, National Center for Scientific Research “Demokritos”, Patr. Gregoriou Ε’ and 27 Neapoleos Str., Aghia Paraskevi, 15341 Athens, Greece; d.kefallinou@inn.demokritos.gr; 2 Laboratory of Inflammation and Autoimmunity, Biomedical Research Foundation, Academy of Athens, 11527 Athens, Greece; mgrigoriou@bioacademy.gr (M.G.); dboumpas@bioacademy.gr (D.T.B.); 3 4th Department of Internal Medicine, Attikon University Hospital and Joint Rheumatology Program, National and Kapodistrian University of Athens, 12462 Athens, Greece

**Keywords:** organs-on-chips (OoCs), bone-marrow-on-a-chip (BMoC), 3D microfluidic cell culture devices, microfabrication, porous poly(dimethylsiloxane) (PDMS) membrane, bone marrow organoid, hematopoiesis, hematopoietic stem and progenitor cells (HSPCs), mesenchymal stem cells (MSCs)

## Abstract

Bone marrow has raised a great deal of scientific interest, since it is responsible for the vital process of hematopoiesis and is affiliated with many normal and pathological conditions of the human body. In recent years, organs-on-chips (OoCs) have emerged as the epitome of biomimetic systems, combining the advantages of microfluidic technology with cellular biology to surpass conventional 2D/3D cell culture techniques and animal testing. Bone-marrow-on-a-chip (BMoC) devices are usually focused only on the maintenance of the hematopoietic niche; otherwise, they incorporate at least three types of cells for on-chip generation. We, thereby, introduce a BMoC device that aspires to the purely in vitro generation and maintenance of the hematopoietic niche, using solely mesenchymal stem cells (MSCs) and hematopoietic stem and progenitor cells (HSPCs), and relying on the spontaneous formation of the niche without the inclusion of gels or scaffolds. The fabrication process of this poly(dimethylsiloxane) (PDMS)-based device, based on replica molding, is presented, and two membranes, a perforated, in-house-fabricated PDMS membrane and a commercial poly(ethylene terephthalate) (PET) one, were tested and their performances were compared. The device was submerged in a culture dish filled with medium for passive perfusion via diffusion in order to prevent on-chip bubble accumulation. The passively perfused BMoC device, having incorporated a commercial poly(ethylene terephthalate) (PET) membrane, allows for a sustainable MSC and HSPC co-culture and proliferation for three days, a promising indication for the future creation of a hematopoietic bone marrow organoid.

## 1. Introduction

Human bone marrow is attributed the vivifying role of hematopoiesis, all the while being an essential part of the lymphatic system, contributing to the shielding of the immune system [1]. Red bone marrow specifically, located in the cancellous, spongy bone, is home to hematopoietic stem cells (HSCs) endowed with the unique properties of self-renewal and differentiation into all the vital mature blood cells, such as red blood cells, lymphocytes, and platelets, and other cells (e.g., macrophages, neutrophils) distributed throughout the body via the systemic circulation [2].

HSCs reside in a specialized microenvironment within the bone marrow, alongside their multi-potent progenitors, called the hematopoietic niche. The HSCs and their progeny are referred to as hematopoietic stem and progenitor cells (HSPCs). The niche is a complex structure composed of multiple cell lineages, the extracellular matrix (ECM), and secreted factors, whose coordinated regulation promotes the maintenance, differentiation, and function of the HSCs [3]. Amongst the most important cells for sustaining HSCs are mesenchymal stem cells (MSCs) or bone marrow stromal cells [4], as they affect the migration of HSCs [5] and express several transcription factors (e.g., chemokine, stem cell factor) critical for HSC viability [5,6]. The hematopoietic niche is organized into two main regions: the endosteal niche, located on the surface of the trabecular bone, and the perivascular niche, which surrounds the blood vessels [7].

Considering the significance of hematopoiesis, understanding its association with physiological and pathological processes has attracted a great deal of attention. Conventional biomedical tools, restricted to 2D or 3D static cell cultures and animal testing, are still the cornerstone of studying the physical and biochemical cues of cells and tissues [8,9]. However, cell cultures fail to recreate the dynamics of the cellular microenvironment [10,11], while animal trials are susceptible to interspecies differences [12,13].

Advances in microfluidic technology in coalition with cellular biology have leveled up biomedical research with the advent of organs-on-chips (OoCs). OoCs are 3D microfluidic devices lined with human cells that recapitulate vital functional units of organs [14,15]. OoCs go far beyond by offering with greater fidelity the diversity of the organ-specific microenvironment. They leverage the miniaturization advantages of microfluidic systems, involving better spatiotemporal control and reproduction of the necessary biochemical gradients, flows, and mechanical stresses, to reproduce the cellular microenvironment [16]. This way, intracellular interactions, tissue physiology, and tissue–tissue communication are more faithfully recreated all the way up to the organ level [17,18].

In this context, bone-marrow-on-a-chip devices and, specifically, the on-chip portrayal of the bone marrow hematopoietic niche have become increasingly evolving scientific fields. The majority of the existing works on the constructed BMoC devices focus only on the in vitro maintenance of hematopoiesis, and not on its on-chip generation. This applies to the innovative studies of Torisawa et al. [19,20] and Sieber et al. [21], where, in the first case, the marrow organoid was created in vivo and subsequently preserved in vitro, and, in the second case, the MSC–HSPC co-culture was performed outside the chip and afterwards transferred onto the chip for preservation. In addition, regarding the studies that achieved a combination of in vitro generation and maintenance, there are several that used commercial platforms [22,23], which are integrated systems with more complex geometries and utilities that undoubtedly enable better control of the experimental conditions. Among those that actually constructed chips for both the generation and maintenance of hematopoiesis, they incorporated at least three kinds of cells [24], while others emulated both the perivascular and endosteal areas of the hematopoietic niche using four different cell types [25,26]. Besides these, all the existing BMoC devices, in order to mimic the bone marrow microenvironment, incorporate a hydrogel/gel of natural ECM protein origin (e.g., type I collagen and fibronectin) [19,20,25,26] or a synthetic scaffold usually made out of hydroxyapatite or polystyrene and generally with a bone-like structure (e.g., ceramics) [21]. Both gels and scaffolds help to create 3D structures to support the hosting of HSCs.

Herein, a BMoC device that is intended for the purely in vitro generation and maintenance of the hematopoietic niche without the incorporation of gels or scaffolds is introduced. Our goal was to simulate the bone marrow hematopoietic niche close to the perivascular region, where there are no vessels and the HSPCs are in a more quiescent state compared with the core of the perivascular region. It is the region where the biological regulation of HSCs is performed. Specifically, only two types of cells, MSCs and HSPCs, were used, which constitute a simplified biological model that is, however, sufficient to simulate the region close to the perivascular niche on-chip. Moreover, unlike the existing studies that involve gels or scaffolds, this work pioneers the use of only living cells to create the bone marrow niche, allowing them to produce all the necessary cytokines and factors for their interaction. Specifically, we advocate that the MSCs secrete the growth factors and cytokines necessary for the creation of a 3D matrix bearing the appropriate chemical and mechanical properties to support HSPCs [27]. After all, the perivascular region, unlike the endosteal region, is not characterized by high stiffness, meaning that the absence of solid, condensed scaffolds does not hamper its faithful mimicry.

More specifically, the BMoC described herein is a poly(dimethylsiloxane) (PDMS)-based device that consists of a cell culture microchamber, a medium reservoir, and an intervening porous membrane, which is either a constructed PDMS membrane or a commercially obtained poly(ethylene terephthalate) (PET) membrane. It should be highlighted that a unique methodology for the PDMS membrane’s fabrication is introduced, which, unlike many studies [28,29,30], involves in-house realization of all the production stages and, most importantly, leads to reproducible, successful results. We proceed with the fabrication steps of the BMoC device, from its design to its final construction. Of note is that the BMoC is passively perfused, in the sense that it is not actively perfused with a pump, but at a lower rate via medium diffusion from a large-capacity culture dish. This is to address the everlasting problem of bubble formation, which is inherent to long-term perfused microfluidic systems [31] and is particularly damaging in OoC devices, where cell survival is hampered at the liquid–gas interface [32,33]. All the existing BMoC studies avoid any reference to bubble accumulation and most probably resolve it with the utilization of bubble traps.

Furthermore, the two membranes (the PDMS and PET membranes), as part of the chip, were tested regarding their ability to support MSC development. Finally, MSCs and HSPCs were co-cultured inside the passively perfused BMoC for three days in order to primarily assess their co-existence and their distribution during the co-culture and, specifically, to examine whether the HSPCs were uniformly organized within the created stromal tissue. This information was obtained with fluorescence measurements, proving the sustainable co-culture and proliferation of both cell lineages. The MSCs appeared to create a 3D microenvironment that supports HSPC growth. Hence, the passively perfused BMoC, purely in vitro and without auxiliary 3D structures (gels/scaffolds), successfully sustained the MSC–HSPC co-culture, which is an optimistic outcome toward the future creation of a hematopoietic bone marrow niche. Prolongation of the co-culture to a week’s time is sought in order to examine organoid formation.

## 2. Materials and Methods

### 2.1. Chip Design and Fabrication

**Figure 1:**
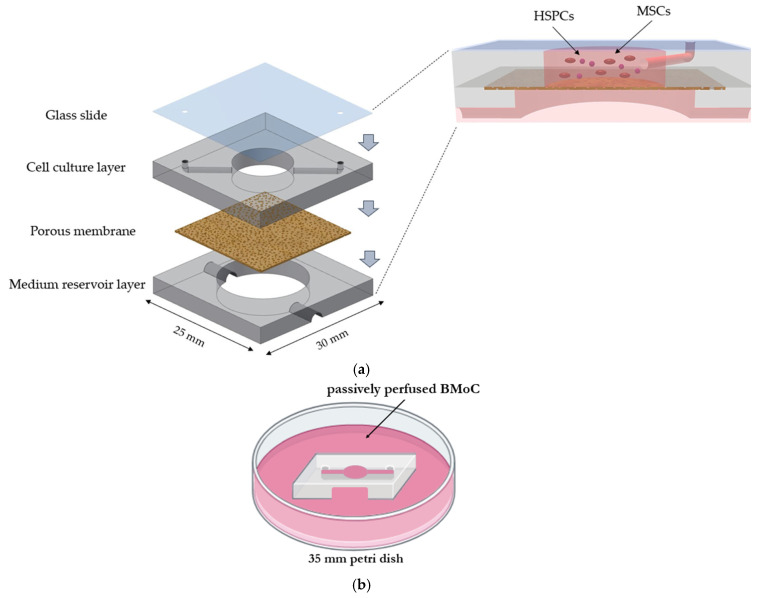
Schematic representation of the passively perfused bone-marrow-on-a-chip (BMoC) device. (**a**) Exploded 3D illustration of the chip, consisting of two PDMS layers, the upper cell culture microchamber and the lower medium reservoir, separated by a porous membrane. The device is sealed on the top with a glass slide for cell observation. In magnification, a cross-section of the chip is presented, showing the mesenchymal stem cell (MSC) and hematopoietic stem and progenitor cell (HSPC) co-culture in the microchamber as well as the inserted interconnections reaching the microchamber. (**b**) Three-dimensional representation of the chip immersed in a 35 mm culture dish filled with medium. Passive perfusion of the chip is ensured via diffusion from the dish to the cell microchamber through the porous membrane.

The passively perfused BMoC device comprises two layers: an upper one forming the cell culture microchamber and a lower one serving as the medium reservoir, while their interconnection is ensured through a porous membrane. Both the top culture layer and the reservoir layer are made of PDMS (Sylgard 184 silicone elastomer kit, Dow Corning, Midland, MI, USA), while the membrane is fabricated out of either PDMS or commercially supplied track-etched poly(ethylene terephthalate) (PET) (it4ip S.A., Ottignies-Louvain-la-Neuve, Belgium). In particular, the PET membrane has a 0.4 μm pore size, with the pores being aligned in parallel, a pore density of 4 × 10^6^ pores/cm^2^, a porosity of 0.5%, and a permeability of 144 mL/min/cm^2^ at 0.7 bar. The chip is sealed at the top with a glass slide, through which cell observation is performed. Figure 1a shows a 3D schematic representation of the chip layers as well as a cross-section of the chip, illustrating the MSC and HSPC co-culture in the upper microchamber. Both the cell culture microchamber and the reservoir are of a cylindrical shape, with the reservoir being additionally transparent and remaining submerged in a 35 mm culture dish filled with medium. Figure 1b presents schematically the chip immersed in the dish.

The chip was fabricated mainly by using the replica-molding technique [34] for the construction of the microchamber and the reservoir, as already reported elsewhere [35]. For the microchamber, 3D computer-aided design (CAD) software (Autodesk Fusion v. 2.0.18950) was used to design its mold, which was later realized in an aluminum substrate with Computer Numerical Control (CNC) micromachining. PDMS at a 10:1 (base:agent) volume ratio was cast against the mold and left to cure at 100 °C for 40 min. This way, the resulting PDMS microchamber bears the relief structures of its mold. As for the reservoir, after PDMS curing, it was opened with an 8 mm biopsy punch.

The microchamber is 6 mm in diameter and 0.5 mm thick, while the reservoir is 8 mm in diameter and 1.5–2 mm thick. The microchamber and reservoir PDMS layers, as well as the glass slide, are of common dimensions (30 × 25 mm^2^ length × width).

For chip assembly, the PDMS layers and the glass slide are exposed to air plasma treatment in a low-pressure Covance multi-system (Femto Science Inc., Hwaseong-si, Republic of Korea) that has already been proven to induce PDMS surface oxidation [36]. The activation conditions were 100 W, 5 × 10^−^^2^ Torr (8.27 × 10^−^^1^ Torr during the process), 50 sccm, and 1 min.

The surface properties of the plasma-treated PDMS surfaces were assessed through water static contact angle (WSCA) measurements using the DSA30 Drop Shape Analyzer (KRÜSS GmbH, Hamburg, Germany). In brief, a 2 μL sessile droplet of deionized water was placed on the surface, and the static contact angle was determined through tangent fitting. Each surface was tested in triplicate, with at least two measurements taken at different spots on each surface. The results are reported as average values with their ± standard error.

The bonding process is slightly differentiated depending on the kind of membrane used. In any case, the process is serial, and the less flexible a material, the later it is bonded. Specifically, when the PDMS membrane is incorporated, it is the first one to be bonded with the cell microchamber following their union with the reservoir. Regarding the PET membrane, the microchamber is bonded with the reservoir, and the membrane is simply held in between. In both cases, the glass slide is bonded at the end. Finally, holes with a diameter of 1–1.3 mm are drilled into the glass, in alignment with the inlet and outlet of the microchamber, to allow for the insertion of interconnections (Figure 1a, in magnification).

The BMoC device was inspected with scanning electron microscopy (SEM) using a JSM-7401F field-emission microscope (JEOL, Tokyo, Japan).

### 2.2. PDMS Membrane Fabrication and Characterization

The process for the construction of a porous PDMS membrane is described in our previous work [35]. It is based on the protocol by Huh et al. [37], which entails the pressing of a PDMS carrier, bearing a thin PDMS coating of the desirable membrane thickness, onto a micropillar arrayed Si mold for boring holes into the PDMS, thereby leading to porous PDMS membrane formation.

The Si mold is constructed by negative tone photolithography (AZ4562 resin, MicroChemicals GmbH, Ulm, Germany) for the transfer of the microarray to Si, followed by the deep reactive-ion etching (DRIE) Bosch process of a sulfur hexafluoride (SF_6_) and octafluorocyclobutane (C_4_F_8_) gas mixture for the fabrication of Si micropillars. The Bosch process was conducted in a high-density inductively coupled plasma (ICP) reactor (Micromachining Etching Tool, Adixen-Alcatel, Anaheim, CA, USA) at previously reported conditions [35] (1800 W, −70 V bias, 15 °C, 40 mTorr, 171 sccm SF_6_/115 sccm C_4_F_8_, and 7 s/3 s timing steps).

Thus, according to the protocol, a PDMS pre-polymer of a 3:1 (base:agent) mixing ratio is spin-coated at a thickness of about 20 μm on a completely cured 15:1 ratio PDMS carrier of 2.5 × 2.5 cm^2^ and 5 mm thickness. The coated carrier is then reversed upon the 10 × 10 mm^2^ micropatterned Si mold and conformally pressed with a 45 g aluminum plate to allow the pillars to penetrate the pre-polymer. The entire stack is afterwards left at room temperature for 30–40 min, and subsequently at 100 °C for approximately 2.5 h, to allow for complete curing of the pre-polymer. The carrier with the now-cured PDMS is later removed from the mold, resulting in the creation of a porous PDMS membrane on its carrier.

PDMS membrane observation was carried out using SEM (JEOL JSM-7401F microscope, Japan).

### 2.3. Cell Isolation and Culture

Umbilical-cord-derived mesenchymal stem cells were donated from the “Hellenic Cord Blood Bank” (H.C.B.B., Athens, Greece), which performed all the isolation and culture steps up to cell passage 1. MSCs are stored until use in freezing medium (90% FBS, 10% DMSO) and stored at −80 °C. They are usually seeded at a 1:100 dilution in a T75 flask with 10–12 mL of the alpha modification of Eagle’s Minimum Essential Medium (αMEM, Gibco/22571–020, Carlsbad, CA, USA) containing 1% penicillin–streptomycin (Gibco/15140–122, Carlsbad, CA, USA) and supplemented with 20% fetal bovine serum (FBS, Gibco/10270–106, Carlsbad, CA, USA) (heat-inactivated) and 1% L-glutamine. The medium is renewed every 3–4 days, whenever needed. When cells reach 80–90% confluence, they are harvested using 1× trypsin/EDTA (7 min, 37 °C), centrifuged (1200 rpm, 7 min, 25 °C), and re-plated. MSCs are kept in culture until passage 8.

HSPCs (CD34^+^) were isolated from human bone marrow mononuclear cells (BMMCs) (adult samples) with magnetic beads (StemCell Technologies/18056, Vancouver, BC, Canada). CD34^+^ cells were cultured in 96-well plates with StemSpan™ H3000 medium (StemCell Technologies/09850) and 1% penicillin–streptomycin (Gibco, 15140) supplemented with the cytokines (100 ng/mL) TPO (Immunotools/11344863, Friesoythe, Germany), (1/400), SCF (Immunotools/11343323), and Flt3-Ligand (Immunotools/11343303).

Cells were kept in a humidified incubator (Forma™ Steri-Cycle™ CO_2_ Incubator, Thermo Scientific™, Waltham, MA, USA) providing the appropriate conditions (5% CO_2_ supply, 37 °C). They were observed under a Leica DM IRE2 inverted light microscope (Leica Microsystems, Wetzlar, Germany). All the mentioned culture reagents were purchased from Thermo Fisher Scientific (Waltham, MA, USA).

### 2.4. On-Chip Cell Culture

After 30 min of UV sterilization of the chip, its cell culture microchamber was filled with type I bovine collagen coating solution (50 μg/mL, Sigma-Aldrich, Taufkirchen, Germany) and incubated at 37 °C for 30 min. Application of the collagen solution to the plasma pre-treated PDMS surfaces has been proven to promote collagen immobilization [36]. The chip was temporarily placed inside a poly(methyl methacrylate) (PMMA) holder and sealed with screws to reduce solution evaporation. Later, to remove the unbound solution, the microchamber was rinsed twice with serum-free medium. The chip holder was removed, and the chip was set for cell seeding.

In the case of the PET–PDMS membrane comparison study, MSCs formerly stained with DiO (green) cell-labeling solution at a 1:100 dilution ratio were inserted at an initial concentration of 5.6 × 10^5^ cells/mL into each chip. The chips were submerged in different culture dishes filled with MSC culture medium and maintained inside the humidified incubator with 5% CO_2_ at 37 °C for 3 days.

Cell observation was performed daily using the Leica inverted microscope for both optical and fluorescence imaging. The thickness of the formatted 3D stromal tissue inside the chips was measured by optical observation. By displacing the focal plane at the upper and lower boundaries of the tissue, the apparent tissue thickness was estimated (d_apparent_). In order to estimate the actual tissue thickness (d_actual_), a correction is needed, taking into account the light refraction through the medium as well as the depth of field (DOF) of the lens, according to the formula

(1) $$d_{\mathrm{actual}}=(d_{\mathrm{apparent}}-\mathrm{DOF})\frac{n_{\mathrm{medium}}}{n_{\mathrm{air}}},$$

where the DOF = 11.5 μm for 10× magnification, the medium refractive index, n_medium_ = 1.4, and the air refractive index, n_air_ = 1.

Analysis of the fluorescence images was carried out with ImageJ software (v. 1.46r) to yield the cell spread area (μm^2^) inside the microchamber as a result of at least 5 images from each chip. A 3D surface tissue plot, which presents the stromal tissue distribution on each membrane, was derived using the same software from characteristic fluorescence images of each chip.

Further, image analysis of the same fluorescence images used for the 3D surface tissue plots on the PDMS and PET membranes was performed to quantify the uniformity of the tissue distribution and, therefore, make more evident the differences in the membranes. First, the histograms of the fluorescence intensities in each image were calculated for the PDMS and PET membranes. In order to elucidate more the uniformity differences between the PDMS and PET membranes, we proceeded to a more detailed analysis of the spatial distribution of the pixels in the fluorescence images. To this end, the fluorescence images of each membrane were divided into a mesh of square boxes with a specific side length that was much larger than the pixel size. Then, the mean intensity of the pixels belonging to each square box was calculated and, from that, the standard deviation (or the variance) of the mean intensity of the boxes was calculated. The spatial uniformity of the fluorescence images was quantified by the graph of the standard deviation versus the number of square boxes required to cover the entire image for both the PDMS and PET images.

For the MSC–HSPC co-culture, the chip with the PET membrane was first seeded with DiO (green)-stained MSCs at a 6.4 × 10^5^ cells/mL concentration that had been cultured for 2 days. On the 3rd day, when the MSCs reached 80% confluence, HSPCs at a 2.5 × 10^5^ cells/mL initial concentration were added and remained in the co-culture for 3 days. The HSPCs had also been previously stained with DiI (red) cell-labeling solution (at a 1:100 dilution ratio) before entering the chip. The chip was submerged in a culture dish containing StemSpan™ H3000 medium enriched with cytokines (IL-3, SCF, Flt3-Ligand, and G-CSF, each at a concentration of 100 ng/mL) and was incubated in a humidified atmosphere of 5% CO_2_ at 37 °C. The Leica inverted microscope was used for imaging, and the ImageJ software was used for the estimation of the % MSC area on the top tissue layers, as well as the HSPC spread area, until the 3rd day. At least 5 fluorescence images were used for the area calculation.

In any case, the plate’s medium was renewed manually every 1–2 days depending on the needs of the culture.

## 3. Results and Discussion

### 3.1. Membrane Fabrication

Following the abovementioned protocol and conditions, a mechanically strong porous PDMS membrane was successfully fabricated. The membrane totally detached from the Si mold and remained undisrupted on the PDMS carrier. Following this, at the bonding stage, the membrane was completely detached from its carrier to be bonded with the cell microchamber.

**Figure 2:**
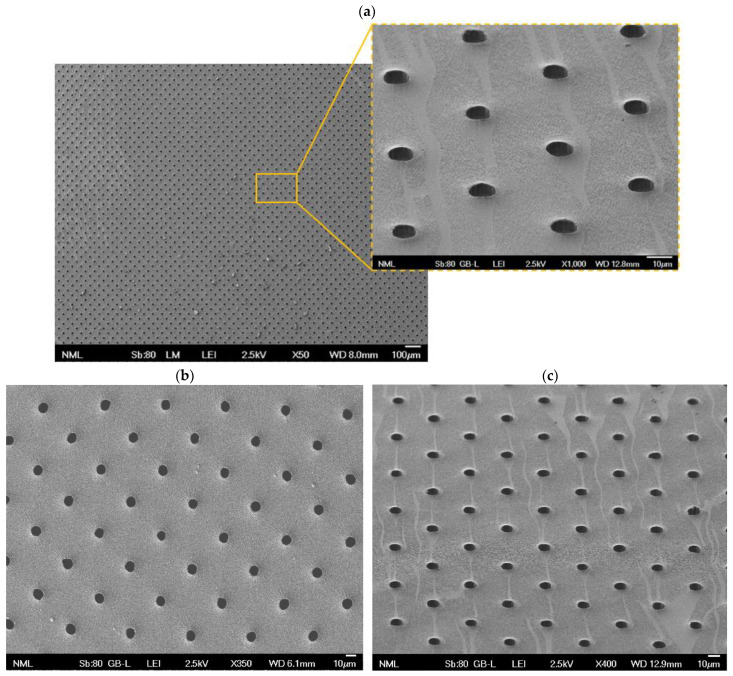
Scanning electron microscopy (SEM) images of the constructed porous PDMS membrane. (**a**) Top-down 50× magnification image and an under 60° tilt 1000× magnification image, where almost 100% opening of the pores can be observed. (**b**) Top-down image at 350× magnification, from which the average pore diameter was estimated to be 10.2 ± 0.2 μm. (**c**) Image at 400× magnification under 60° tilt, where the spotted regions of lighter color are associated with CF_x_ remnants deriving from the membrane’s manufacturing process.

SEM was used to examine the produced membrane from the back side, the one previously in contact with the carrier, to ensure pore opening. To begin with, Figure 2a–c clearly demonstrate the fabrication of an intact membrane. Top-down observation (Figure 2a, 50× magnification) showed that almost 100% of the pores were thoroughly open, which was also confirmed by a 60° tilt observation (Figure 2a, 1000× magnification), where the pores appear to have depth. The average pore diameter, as derived from different areas of images at 350× magnification (Figure 2b), was found to be 10.2 ± 0.2 μm, while the center-to-center pore distance was 40 μm. Moreover, as seen in Figure 2a (1000× magnification) and Figure 2c at a 60° tilt, regions of lighter coloration can be spotted on the membrane. These can most probably be attributed to CF_x_ residues of the fluorocarbon hydrophobic layer from trifluoromethane (CHF_3_) plasma deposition on the PDMS carrier that were transferred to the membrane when in contact. It should be noted that the area and distribution of the spotted regions appearing in Figure 2a,c are indicative and might differ on other membrane parts or other membrane fabrication trials.

PDMS is one of the most dominant materials for OoC membranes [38,39] given its innate assets, such as transparency, gas permeability, and flexibility [40,41], the latter being especially well-suited for applications that require mechanical stretching of the membrane [42,43]. It is an undeniable truth that, in many OoC studies, the porous membrane, made out of either PDMS or another polymer (e.g., PET or poly(carbonate) (PC)), has been purchased in the first place [44,45,46,47]. Regarding the PDMS membrane’s fabrication, various novel methods have been registered, among which are electrospinning [48], pore formation via high-pressure saturated steam [49], and 3D printing for more complex geometries [50,51]. However, the most traditional PDMS membrane microfabrication methods are still preferred specifically for membrane thicknesses equal to or greater than 10 μm [38]. These methods conform to one of the two main protocols introduced by Kim et al. [16] and Huh et al. [37], which greatly rely on the existence of a microfabricated mold and replica molding. A variety of studies follow the protocol of either Kim et al. [28,52] or Huh et al. [29,30,53], and a large number involve a commercially attained microfabricated mold [28,29,30].

Our work is one of the few studies where all stages of the membrane’s fabrication were produced right from the beginning. Moreover, even though the protocol’s steps were adopted from Huh et al. [37], we have proposed a unique manufacturing methodology for porous PDMS membrane fabrication, whose success is based on the rationale that the chemical passivation of the mold ought to be stronger than the one of the carrier in order to enable membrane detachment from the mold and adherence to the carrier [35].

On the whole, a porous PDMS membrane was effectively constructed. It should also be highlighted that the suggested methodology is reproducible and has led to the successful production of at least five membranes.

### 3.2. Chip Fabrication

As mentioned before, bubble formation is an innate phenomenon of microfluidic devices under flow, with detrimental side effects in cell-related applications, as is the case for organs-on-chips. However, in addition to cell survival and proliferation, the nutrient media supply in organs-on-chips is essential in order to better recapitulate the dynamics of the cellular microenvironment up to the organ level [17,18]. Addressing both issues, we introduced a passively perfused BMoC device, which is not actively perfused, using for example a pump, but is submerged in a large-capacity culture dish, where the perfusion occurs naturally via diffusion from the dish to the cell microchamber through the porous membrane. This way, we manage to both eliminate bubble insertion and additionally sustain dynamic medium renewal, except at a lower rate. In addition to these, bone marrow cells are not naturally exposed to high mechanical stress or large gradients of pressure, velocity, and concentration as happens with other organs, for instance the lungs [54], heart [55,56], and intestine [16,57], that present inherent motion. Thus, nutrient media supply at a lower rate with the passively perfused BMoC device does not hinder the creation and maintenance of the bone marrow organoid.

When evaluating the device’s integrity and functionality, having produced the constituent PDMS layers with replica molding, the bonding series using air plasma treatment, as reported in Section 2.1, was found to have resulted in successful sealing. This comes to terms with the PDMS wettability testing after the plasma treatment, which registered an average value of 16.3° ± 1.3°, validating surface activation. Plasma treatment during this bonding process acts as the essential PDMS surface activation step to render it hydrophilic. Surface wettability is of paramount importance to cell adhesion and proliferation, since hydrophobic surfaces act in an inhibitory manner [58,59]. As PDMS is inherently hydrophobic (112.9° ± 0.2°), the induced surface activation after the plasma treatment successfully manages to increase its surface energy.

When using the PDMS membrane, the presence of CF_x_ residues on it (see Section 3.1) did not compromise its bonding with the reservoir. Accordingly, with the PET membrane, the irreversible bonding between the two PDMS layers, the cell microchamber and the reservoir, is sufficient to ensure the grasping of the membrane in between and, therefore, the sealing of the chip.

**Figure 3:**
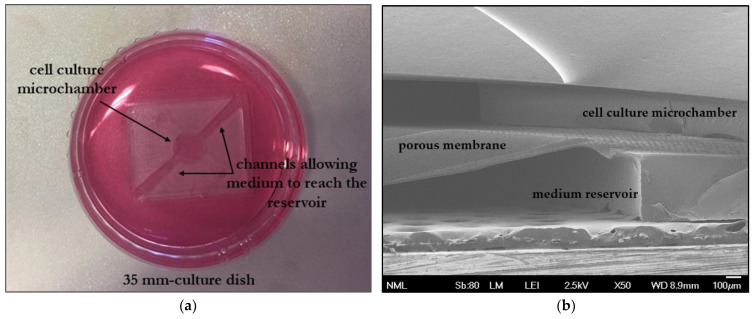
The passively perfused BMoC device. (**a**) Photo of the device submerged in a 35 mm culture dish during the experimental process. Diametrically opposed channels were opened to allow for the medium’s replenishment in the chip reservoir. (**b**) SEM image (50× magnification) of a cross-section of the constructed BMoC device demonstrating its PDMS constituents, the cell culture microchamber, the medium reservoir, and the intervening PDMS membrane.

Moreover, since the device was submerged in a culture dish, after sealing, channels (up to 2 mm wide) were opened with a scalpel diametrically in the reservoir to ensure the medium’s replenishment in the reservoir. Figure 3a shows a realistic photo of the constructed BMoC device immersed in a culture dish filled with medium, where the channels are specifically indicated with arrows.

Overall, the fabrication process of the chip, including either type of membrane, was reproducible and led to the successful production of multiple chips. In Figure 3b, a cross-section of the BMoC device under SEM is demonstrated, showing the cell culture microchamber and medium reservoir with the intervening porous PDMS membrane. A completely functional, passively perfused BMoC device has, therefore, been reliably constructed.

### 3.3. MSC Culture on the PET and PDMS Membrane in the Passively Perfused BMoC

**Figure 4:**
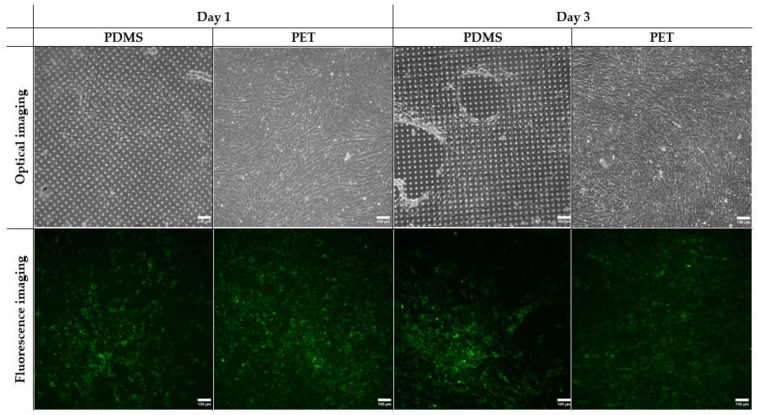
Comparison of MSC growth under microscope inspection on the porous constructed PDMS and the commercial PET membranes inside the passively perfused BMoC on the 1st and 3rd day of culture. The optical and fluorescence images were captured on different membrane regions. Both the optical and fluorescence images (10× magnification) present more uniform cell spreading on the PET membrane. Voids in stromal tissue regions can be spotted on the optical images, indicated as dark areas in the fluorescent images.

BMoC devices embedded with a commercial PET membrane or the constructed PDMS membrane were compared in terms of their ability to sustain the MSC culture. MSCs stained with DiO solution were seeded at a concentration of 5.6 × 10^5^ cells/mL on the chips and cultured for 3 days. Optical and fluorescence microscopy were performed to evaluate cell expansion, representative images of which on the first and third day are shown in Figure 4.

In general, it is evident from all images that MSCs appear to be more uniformly organized on the PET membrane. This is obvious even from the first day of optical imaging, when they have expanded to the largest part of the PET membrane. MSCs have maintained their spindle morphology and have already managed to form stromal tissue with locally circular routes. On the third day, their development leads to complete confluence and a consistent, uninterrupted tissue network.

However, this is not the case for the PDMS membrane, where MSCs are not distributed uniformly; rather, they tend to coalesce, leaving several areas uncovered, as can be seen on the first day. Because of that, many of them lose their fibroblast-like shape, ending up regionally accumulated. Over the next few days up to the third day, MSCs systematically avoid spreading in these specific areas, preferring to grow vertically instead. This way, uneven stromal tissue is formed, whose circular orientation is disrupted by void areas. MSCs appear to specifically pile up on the periphery of those voids. Tissue thickness measurements via optical observation were performed on the thrid day according to Equation (1), which resulted in a thickness of 74.4 ± 3.6 μm on the PDMS membrane, compared with 59.5 ± 5.8 μm on the PET membrane on the third day. Therefore, the locally larger tissue thickness on the PDMS membrane verifies the vertical MSC accumulation.

All these observations are consistent with the fluorescence images. In particular, while uniform spreading of the labeled MSCs on the PET membrane occurred on the first and third day, a clear distinction between the fluorescent and the dark areas can be made on the PDMS membrane. In fact, this is more pronounced on the third day with the increase in the MSC population. In places, a higher cell density appears on the PDMS membrane compared with the PET membrane as a result of cell accumulation and the avoidance of specific areas, which persistently appear dark in color.

**Figure 5:**
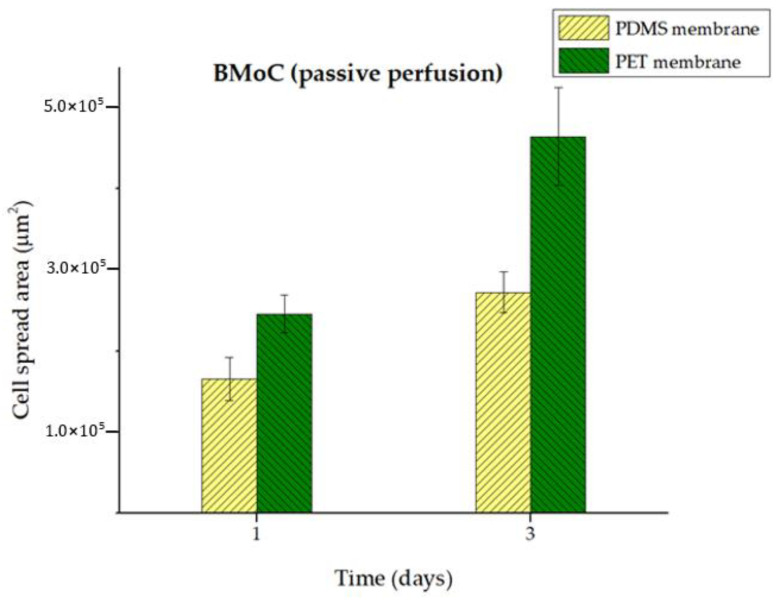
Average area covered by MSCs (μm^2^) on the constructed PDMS and the commercial PET membranes inside the passively perfused BMoC on Days 1 and 3. Although both membranes present an increase in cell coverage area from Day 1 to Day 3, the PET membrane shows a higher degree of MSC spreading, almost twice as much as the PDMS membrane on Day 3.

Analysis of the fluorescence images was used to estimate the cell spread area on both membranes on the first and third days (Figure 5), taking into account at least five images in every case. The results are presented as the average spread area alongside its standard error. It should be noted that the calculated area values are lower compared with those observed in real time, since the fluorescence of the lower tissue levels is not always included. Thus, the average MSC spread area on the first day was 1.7 × 10^5^ μm^2^ on the PDMS membrane and 2.5 × 10^5^ μm^2^ on the PET membrane, i.e., approximately 1.5 times bigger on the PET membrane. On the third day, the PDMS membrane yielded an average area of 2.7 × 10^5^ μm^2^, compared with 4.6 × 10^5^ μm^2^ on the PET membrane, which is an increase of nearly 1.7 times on the PET membrane. These values correspond to cell coverage percentages of 15% and 26% for the PDMS and PET membranes, respectively. The greater surface spread of MSCs on the PET membrane is confirmed.

Focusing on the identified tissue disruptions on the PDMS membrane, they are probably due to the presence of CF_x_ residues on it, as mentioned in Section 3.1 (Figure 2a,c), originating from the deposition of the hydrophobic CHF_3_ layer on the PDMS carrier, with which it remained in contact. These residues, varying in size and distribution, jeopardize collagen immobilization and, thereby, cell attachment, since fluorocarbon groups (-CF_x_) are known to prevent cell adhesion [60]. This justifies the existence of specific PDMS membrane areas that steadily remained uncovered with stromal tissue throughout the days.

**Figure 6:**
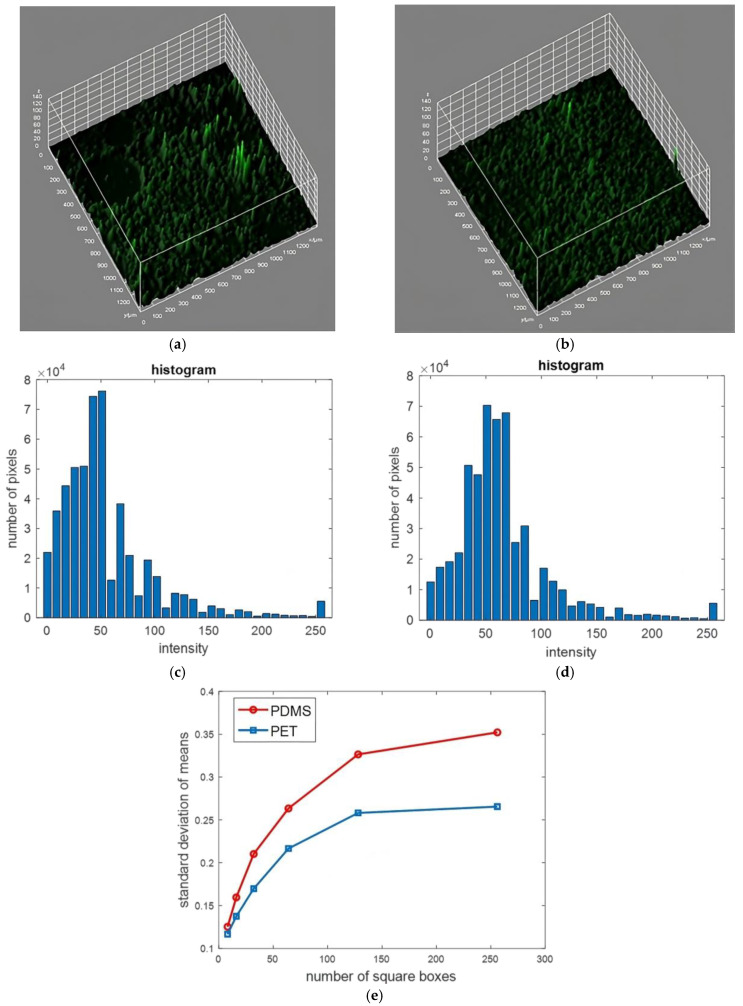
Three-dimensional surface tissue plot of the MSC tissue distribution inside the BMoC on the porous (**a**) PDMS membrane and (**b**) PET membrane on Day 3, based on the corresponding characteristic fluorescence images. On the PDMS membrane, extensive dark areas, indicative of the absence of stromal tissue, and locally greater tissue height variations can be spotted due to the hindering of MSC expansion as a result of the PDMS membrane manufacturing process. In contrast, fluorescent tissue can be observed on almost the entire surface of the PET membrane as a straightforward proof of the uniform MSC growth on it. A histogram of the fluorescence intensity in images of (**c**) the PDMS membrane and (**d**) the PET membrane. Darker pixels to the left of the peak in the PDMS membrane indicate more regions left uncovered with tissue. (**e**) Graph of the standard deviation versus the number of square boxes covering the image for the PDMS and PET membranes. The PET image shows a lower standard deviation, signifying a higher degree of tissue uniformity.

Indicatively, Figure 6 shows the 3D surface tissue plot on both membranes on the third day based on representative fluorescence images. Extensive circular dark regions, at least two in number, can be spotted on the PDMS membrane (Figure 6a), in contrast to the PET membrane (Figure 6b), where almost the whole surface fluoresces. In addition, the fluorescent areas on the PDMS membrane show greater height variations compared with the PET membrane, indicative of the inhomogeneous growth of MSCs and their vertical expansion.

The fluorescence images used for the 3D surface tissue plots on the PDMS and PET membranes were used to better characterize the uniformity of the tissue distribution. The fluorescence intensity of each image for the PDMS and PET membranes resulted in the histograms of Figure 6c and Figure 6d, respectively. It seems that, in the case of the PDMS membrane, there are more pixels with low intensities to the left of the peak in histogram plots, validating our allegations of more regions left uncovered with stromal tissue.

To further illustrate the tissue uniformity on the PDMS and PET membranes, Figure 6e presents the graph of the standard deviation as a function of the number of square boxes covering the whole image for both the PDMS (red line) and PET (blue line) membranes. The spatial uniformity of fluorescence images can be quantified by the standard deviation (or the variance) of the mean intensity of the boxes. Large values of the calculated standard deviation indicate less-uniform images since we have boxes with a wide range of intensity levels. In order to justify the uniformity differences at different scales, we can repeat the above calculation for a large spectrum of box side lengths. If the result remains unaltered, then we can conclude safely about the differences in image uniformity. We can notice that, for all numbers of square boxes, the standard deviation of the PDMS image is larger than the standard deviation of the PET image. This result justifies in a quantified manner the increased nonuniformity of the PDMS membranes compared with the PET ones.

Overall, the existence of nonuniformities on the PDMS membrane as part of its manufacturing process, combined with the previous observations on MSC growth, lead us to the preference of the PET membrane to be incorporated into the BMoC device.

### 3.4. MSC–HSPC Co-Culture on the Passively Perfused BMoC

**Figure 7:**
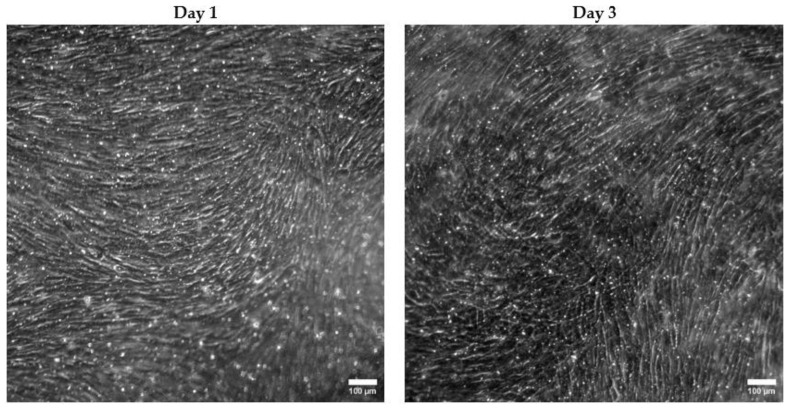
Representative optical microscope images (10× magnification) of the MSC–HSPC co-culture on the first and third day. MSCs appear to be in complete confluence.

**Figure 8:**
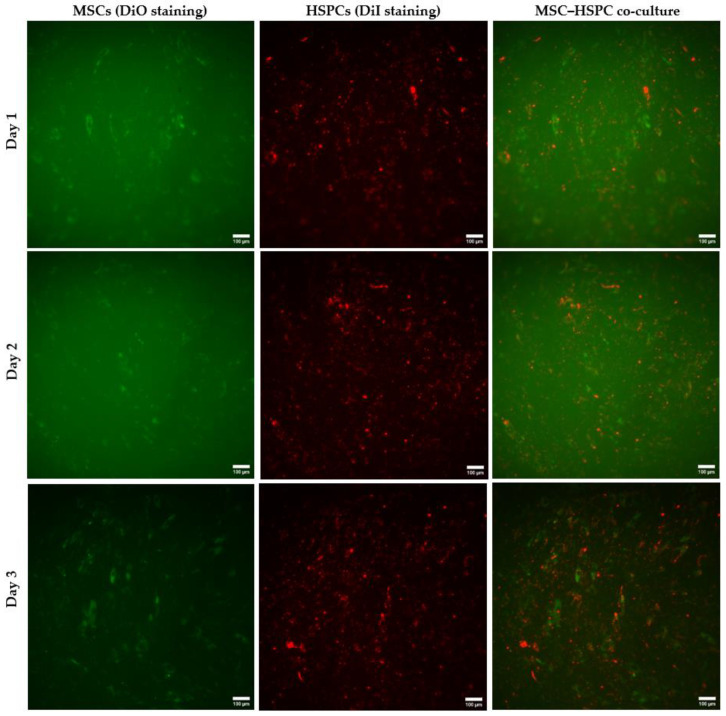
Fluorescence images of the MSC–HSPC co-culture (10× magnification) from the first to the third day inside the passively perfused BMoC. MSCs and HSPCs are presented separately, along with their merged fluorescent outcome. Both MSCs and HSPCs appear to increase their population over the days, as an indication of a sustainable co-existence, which provides optimism for the future creation of an organoid. Scale bar is 100 μm in all images.

The MSC and HSPC co-culture was tested in the BMoC device incorporating the PET membrane. All the MSC and HSPC culture steps were entirely performed on-chip. The MSC culture proceeded at an initial concentration of 6.4 × 10^5^ cells/mL for two days, followed by HSPC seeding on the third day at a 2.5 × 10^5^ cells/mL concentration and a co-culture study for three days. Both the MSCs and HSPCs were labeled with the DiO dye and the DiI dye, respectively, for fluorescence measurements. Microscopy was implemented to capture optical (Figure 7) and fluorescence (Figure 8) images of the co-culture throughout the days.

Optical imaging (Figure 7) on Days 1 and 3 clearly reveals 100% MSC confluence even from the first co-culture day, while the HSPCs cannot be easily distinguished.

**Figure 9:**
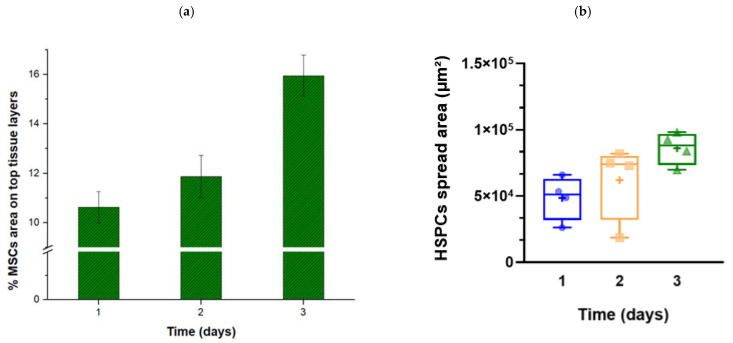
Analysis of fluorescence images of the three-day MSC–HSPC co-culture. (**a**) Percentage MSC coverage of the upper tissue layers over the days. An increase in coverage area can be observed, which demonstrates the gradual spreading of MSCs in the upper layers. (**b**) Representation of the HSPC spread area values (μm^2^) up to the third co-culture day. HSPCs smoothly increase their population over time, showing that the MSCs create a microenvironment capable of sustaining HSPC growth. (*n* = 5, number of dots/triangles shown in the graph).

Moving on to the fluorescence images for quantitative results, Figure 8 displays separate images of the MSCs, the HSPCs, and, finally, their merged outcome of the co-culture from the first to the third day. MSCs appear as spindle-shaped fibroblast-like cells, which is the typical, expected morphology of UC-MSCS, as reported elsewhere [61]. Compared with Figure 7, which certifies the complete confluence of MSCs, the fluorescence images of Figure 8 underestimate the MSC population. The DiO (green) cell-labeling solution, used here for MSC labeling, is a lipophilic fluorescent stain that labels cell membranes and other hydrophobic structures. Thus, when cells proliferate, the dye is expected to dilute and probably fade when there is increased proliferation, as happens in our case. In fact, the fluorescence intensity from the lower stromal layers was very low, resulting in the capture of MSCs from the higher layers, which, again, were underestimated compared with the real-time optical observations. Therefore, Figure 9a illustrates the percentage of the microchamber area covered by MSCs on the top tissue layers (% MSC area on the top tissue layers), as a result of the average of at least five fluorescence images, for each one of Days 1 to 3. Starting from a surface coverage of about 10% on Day 1, it increases to 16% on Day 3, demonstrating the gradual spread of the MSCs on the upper layers and, consequently, the vertical expansion of the tissue.

Regarding the HSPCs, they appear to be uniformly organized in the stroma, not showing signs of regional accumulation. They also increase their population throughout the days, which signifies that they are in an environment that favors their development, without any stressful conditions. To quantify this, analysis of at least five fluorescence images for Days 1–3 for determining the HSPC coverage area (μm^2^) resulted in Figure 9b. The coverage area shows genuinely increasing behavior, with the average values starting at about 4.9 × 10^4^ μm^2^ on Day 1 and increasing to 8.6 × 10^4^ μm^2^ on Day 3 (an increase of about 76% compared with Day 1). These values correspond to 2.7% and 4.8% coverage for Days 1 and 3, respectively. This proliferation of HPSCs is an indirect indication of their viability, signifying that they reside in a non-stressful environment.

In conclusion, MSCs and HSPCs normally exist in a co-culture inside the passively perfused BMoC. MSCs spontaneously generate the stromal tissue that supports HSPC growth and a uniform distribution. Thereby, the BMoC successfully manages to purely in vitro and without 3D auxiliary structures (gels/scaffolds) support the MSC–HSPC co-culture and promote cell proliferation. These results are rather promising for the creation of a hematopoietic bone marrow organoid. We plan to maintain the co-culture for approximately one week in order to investigate the ability of HSPCs to differentiate and examine the existence of the organoid.

## 4. Conclusions

We have presented a BMoC device that aspires to the purely in vitro generation and maintenance of the hematopoietic niche. It is a PDMS-based device that introduces a simplified biological model with two types of cells, MSCs and HSPCs, for the simulation of the hematopoietic niche close to the perivascular region. It relies on MSCs to spontaneously create the bone marrow stroma for hosting HSPCs, thereby pioneering the non-inclusion of any auxiliary 3D structures, like gels or scaffolds. Chip fabrication is based on replica molding, while a porous PDMS membrane has been successfully constructed by introducing a novel methodology that led to its repeated production. To avoid bubble formation, the chip is passively perfused, with medium renewal occurring at lower rate via diffusion from a culture dish. Moreover, comparison of the on-chip MSC growth on the in-house-built PDMS membrane and on a commercial PET membrane yielded nonuniform cell coverage on the PDMS membrane, possibly due to CF_x_ residues originating from its manufacture, leading to the selection of the PET membrane for use in the BMoC. The MSC and HSPC co-culture was tested on the BMoC, with the MSC insertion preceding the HSPC insertion by two days. The HSPC distribution within the 3D stromal tissue was uniform and both cell lineages proliferated within the 3 days of co-culture. Thus, the passively perfused BMoC holds great promise toward the generation and maintenance of a hematopoietic bone marrow organoid.

## Data Availability

All data analyzed during this study are included in this published article.
